# The Teeth of Invertebrate Animals

**Published:** 1891-08

**Authors:** Alton H. Thompson

**Affiliations:** Topeka, Kansas


### THE TEETH OF INVERTEBRATE ANIMALS.[1]

BY ALTON H. THOMPSON, D.D.S., TOPEKA, KANSAS.

The resources of nature are infinite. The expedients to which she resorts are marvellous and endless in their variety. When new conditions are to be met, her invention is never at a loss, and her capacity for change is boundless. Environments change and corresponding alterations in organs arise to meet the new conditions presented. The life of a species depends upon this power to change to conform to new environments. The law is adaptation or extinction.

In no set of organs—in animal life at least—is this infinite variety of resources, or the capacity for change, or the power of invention, so fully illustrated as in the teeth. Food selection has created a wonderful variety of forms of teeth which have arisen in response to changes in the food environment. Those species which could conform to gradual change survived and transmitted the acquired modifications in the dental apparatus. Those which could not change perished, or escaped to a more favorable food environment. From such causes many variations in the teeth of animals arose in the course of the geological ages, and, taking the living and the extinct species altogether, the number and extent of these

---

[1] Read before the Section of Dental and Oral Surgery in the American Medical Association, held at Washington, D.C., May, 1891.

variations is beyond estimation. The variety presented in the different forms of teeth and masticating apparatus throughout the animal kingdom illustrates and exemplifies the fact that these organs are susceptible of great variation, and that the possibilities of change and the invention of nature are especially marked in these organs.

If vertebrate animals present great variations and many extraordinary forms and interesting extremes in the structure of the dental armature, so also do the invertebrate animals, although these are not so well known. To the dental student the teeth of invertebrates are interesting from the comparative stand-point, and serve to illustrate the remarkable possibilities of dental variation, and help to a better understanding of the principles of the mechanical evolution of the teeth of animals. In such studies, any knowledge is valuable which may contribute, even remotely, to a better understanding of the important organs which we are called upon to preserve, and thus better prepare us for our chosen work. The study of comparative anatomy is of great value for the side-lights it throws upon the teeth of man,—their origin, evolution, mechanical design, etc.

Professor Huxley says ("Anatomy of Vetebrates"), "When invertebrate animals are provided with teeth or masticating organs, the latter are either hard productions of the alimentary mucous membrane, or are modified limbs, as opposed to vertebrate animals, which also usually possess hard productions of the alimentary mucous membrane in the form of teeth; but their jaws are ordinarily parts of the walls of the parietes of the head and have nothing to do with the limbs."

The vertebrate jaw is part of the endo-skeleton,—the invertebrate jaw belongs to the exo-skeleton, as do the teeth of all classes of animals, as illustrated by their embryology.

Mr. W. H. Dall says ("American System of Dentistry"), "Almost every large group of organisms below the vertebrates, until we reach the molluscoidæ and lower radiated animals, exhibits in some of its members one form or another of prehensile or masticatory apparatus connected with the alimentary canal. None of these exhibit true homologies with vertebrate teeth, though some of them present remarkable similarity to the latter in external relation. Throughout the invertebrates the teeth are dermal structures, however much modified, and may consist of calcified connective tissue of horny matter, or of chitin or an allied substance. The teeth and jaws of mollusks, the nippers, mandibles, and settæ

of worms, are composed to a greater or less extent of chitinoid material."

Professor A. S. Packard says ("Standard Natural History"), "Hard bodies serving as teeth occur for the first time in the animal series, in the sea-urchins, where a definite series of calcareous dental processes, or teeth, with solid supports and a complicated muscular apparatus, serves for the comminution of food. Among the worms the organs of mastication for the first time appear in the Rotatoria, where the food, such as infusoria, etc., is crushed and is partly comminuted by the well-marked horny and chitinous pieces attached to the mastax. In most other low worms the mouth is unarmed. In the leech there are three, usually in the annelids, two denticulated, serrate, chitinous flattened bodies situated in the extensible pharynx of these worms, and suited for seizing and cutting or crushing their prey.

"In the higher Mollusks, such as the snails and others, besides one or more broad pharyngeal jaws, comparable with those in the worms, is the lingual ribbon, admirably adapted for sawing or slicing sea-weeds or cutting or boring into hard shells, acting somewhat like a lapidary's wheel; this organ, however, is limited in its action, and in the cuttles, the jaws, which are like a parrot's beak, do the work of tearing and biting the animals serving for food.

"In the Crustaceans and insects we have an approach to true jaws, but here they work laterally, not vertically, as in the vertebrates; the mandibles of the articulates are modified feet, and the teeth on their edges are simply irregularities or sharp processes adapting the mandibles for tearing and comminuting food. The numerous teeth lining the crop of crustaceans and insects serve to further comminute the food, keeping the larger particles back till finely crushed."

Professor Bradley says ("Manual of Comparative Anatomy"), "The lowest forms possess no teeth, except some ciliate infusoria, which have an internal cylinder of parallel rods for the mastication of food. In the Rotifera, the denticles are in the shape of denticulated plates. The Echinodermata have five large teeth placed in the formidable apparatus called 'Aristotle's lantern.' In the Annulosa, the leech is the only member that possesses teeth, the semilunar plates embedded in the muscular walls of the mouth; but the remaining classes have only mandibles and maxillæ, which are very hard and chitinous. Among the Mollusca, the gasteropods possess a strap-like organ, the odontophora, which is studded with teeth. Cephalopods possess horny jaws which move vertically. Some other classes have denticles besides.

"In the Annelida so-called teeth occur in many groups, but partake rather of the nature of jaws than teeth. This group comprises most of the worms, as well as the leeches. Their bodies are divided into more or less well-defined, regular segments, and in general the jaws are on the second or buccal segment, or on a proboscis which is itself on the outer edge of this segment, and may be protruded from the mouth to a considerable distance. They are chitinous, most commonly paired, lateral opposite, of almost infinite variety of forms, resembling in a general way the maxillæ of insects, and mimicking, in miniature, hooks, combs, saws, rasps, claws, etc.

"In the leech the mouth is provided with three lenticular jaws, with the projecting edges finely serrated, having a partly rotary motion about a point central to the three. The medicinal leech has two rows of serrations on each jaw.

"Among the Crustaceans (lobsters, shrimps, crabs, etc.), the maxillary organs are but modifications of entire limbs translated from the locomotive series and set apart as special mouth-organs. In the higher crustaceans, the anterior part of the stomach is provided with certain masticatory appendages or stomacholiths, often termed teeth, though more analogous to a sort of a calcareous gizzard. These consist of several calcareous pieces, moved by appropriate muscles inserted in the membranous wall of the stomach, armed with a smooth medium plate and lateral molar-like organs, whose mimetic resemblance to the molar teeth of some forms of mammalia affords a beautiful illustration of the way, through the selective influence of similar functions, analogous structures may be built up in organs which have no homology whatever. Two smaller points, bicuspid in the lobster, tricuspid in the crab, complete the calcareous apparatus.

"Among the Echinoderms, the sea-urchin has a remarkable apparatus called 'Aristotle's lantern,' which contains what may be fairly regarded as true teeth. It is very complicated in its arrangement, but in essentials consists of five hard, calcareous, wedge-shaped sockets or alveoli, each containing one porcelainous chisel-shaped tooth. The teeth are, like those of rodents, usually worn more on the inner than on the outer side, and therefore in wearing always preserve a sharp edge. The combination of the teeth and alveoli produces a pentagonal cone, the apex being formed by the coming together of the points of the teeth. In life this cone is concealed within the tissues, only the points of the teeth projecting."

Not many of the Mollusca are provided with teeth; the entire group of Acephala (the headless mollusks such as clams, oysters, mussels, etc.) are entirely without head or dental apparatus; and not every one of the Cephalopoda (whelks, snails, periwinkles, etc.) are provided with teeth, but most of them have such organs. When they are found, they are arranged on the "odontophore, a chitinous band upon which the teeth are set, pointing upward and backward like the papilla on a cat's tongue, and it grows out of the radular sac in the floor of the gullet. This is controlled by muscles which draw it backward and forward, or even protrude it, as can be seen in the common wood-snail," in which the buccal mass is pushed forward to seize and cut food. In the snail the number of these teeth is remarkable; twelve thousand to forty thousand have been counted on the saw-like lingual ribbon. It can cut grass or leaves sharply off. As the teeth are worn off the ribbon, it is uncoiled and new teeth are thus brought into use. The upper part of the mouth is lined with a horny substance, against which the sharp-toothed tongue works with a rasp-like motion. The tough leaves of the lily may often be found cut by the snail's lingual ribbon.

"The teeth on the strap-like odontophore are varied and remarkable in shape and size and are difficult to examine, as some of them are very minute and hard to dissect out and study. They are usually composed of a base, a shank or stem, and a cutting edge, the latter simple or variously denticulated. The form of the cutting edge is varied, the carnivorous forms usually having simpler and more claw-shaped teeth. When arranged in rows, as they are in many forms, the middle row is called the median or rachidian teeth, and the lateral rows the lateral or pleural teeth. The latter are usually right and left.

"Sometimes there are teeth outside of the lateral rows, which are called the Uncini, and are flat, plate-like, or slender, spiny teeth. They may be very numerous, as in the vegetable-feeding snails, or wholly absent in other forms."

There is much to be observed about the teeth of snails and their allies, and the field offers a profitable opening for investigation. They are already divided into classes by an elaborate system of arrangement, but much remains to be done in describing varieties.

"The adult perfect teeth vary from nearly transparent to an amber-yellow or reddish-brown, and sometimes the cutting points are black. In any large whelk they are easily seen, and in a large cuttle-fish the radula may be an inch wide. On the other hand, in some small land-snails, where the whole shell is not larger than a pin-head, high powers are necessary to observe them. The highest type in the system of classification is called the Toxoglossal, or arrow-toothed, from their narrow, round form, often barbed, and sometimes hollow to inject poison, as in Bela or Conus.

"Next comes Rachiglossa, having only rachidian teeth, as in the common whelk. The teeth are usually slight and varied, and prettily denticulated on the cutting edge. The next is the Tœnioglossa, bent-toothed, including the greater part of the fresh-water snails. The Ptenoglossa, feather-toothed, are a small group, of which the sealaria is a member. The Riphidoglossa, needle-toothed, comprise a large number of sea-snails, and a few operculated land-snails. The last is Docoglossa, chevron-toothed, and includes the limpets."

Some snails present a pavement-like form and arrangement of teeth which are often of a very pretty pattern, or again a mere hardened mass.

We have thus given briefly the outlines of a study of the teeth of invertebrates, merely to indicate the extent of the subject, and to suggest the interest and attractiveness there is in its pursuit to the naturalist; and in addition to this, to suggest that the study of invertebrate odontology has a positive value to the comparative dental anatomist, from a philosophical stand-point. As a leaf from the great book of nature, it unfolds to us many of her beauties and wonders, and it is also pregnant with suggestions to the dental student who follows his subject out into all its branches. So we find in this branch varieties of form and adaptation to purposes which are not paralleled in the vertebrates. The study of their forms and fitness to perform particular duties is full of interest and surprises, in the fertility of design which nature exhibits.

Of homologies with vertebrate teeth there are few, as the jaws of the articulates work horizontally and those of vertebrates vertically. In the few instances of invertebrates which have vertical jaws, those parts are armed with beaks and the teeth are situated farther back on the odontophore. The teeth of the sea-urchin have true sockets and alveoli, but their arrangement, support, and motion are very different from those of the vertebrates; so that taken altogether the class presents few homologies with vertebrates, or even resemblances to them, and thus affords a variety of illustration that the latter does not supply.

